# Multifunctional thiosemicarbazones targeting sigma receptors: *in vitro* and *in vivo* antitumor activities in pancreatic cancer models

**DOI:** 10.1007/s13402-021-00638-5

**Published:** 2021-09-29

**Authors:** Mauro Niso, Joanna Kopecka, Francesca Serena Abatematteo, Francesco Berardi, Chiara Riganti, Carmen Abate

**Affiliations:** 1 Dipartimento di Farmacia-Scienze del Farmaco, Università degli Studi di Bari ALDO MORO, Via Orabona 4, 70125 Bari, Italy; 2 Department of Oncology, University of Turin, via Santena 5/bis, 10126 Torino, Italy

**Keywords:** Pancreatic cancer, Thiosemicarbazone, Sigma receptors, Caspase 3/7/9, Autophagy, PANC-1 xenograft

## Abstract

**Purpose:**

Association of the metal chelating portion of thiosemicarbazone with the cytotoxic activity of sigma-2 receptors appears a promising strategy for the treatment of pancreatic tumors. Here, we developed a novel sigma-2 receptor targeting thiosemicarbazone (FA4) that incorporates a moiety associated with lysosome destabilization and ROS increase in order to design more efficient antitumor agents.

**Methods:**

The density of sigma receptors in pancreatic cancer cells was evaluated by flow cytometry. In these cells, cytotoxicity (MTT assay) and activation of ER- and mitochondria-dependent cell death pathways (mRNA expression of GRP78, ATF6, IRE1, PERK; ROS levels by MitoSOX and DCFDA-AM; JC-1 staining) induced by the thiosemicarbazones FA4, MLP44, PS3 and ACthio-1, were evaluated. The expression of autophagic proteins (ATG5, ATG7, ATG12, beclin, p62 and LC3-I) was also studied. In addition, the *in vivo* effect of FA4 in xenograft models with and without gemcitabine challenge was investigated.

**Results:**

We found that FA4 exerted a more potent cytotoxicity than previously studied thiosemicarbazones (MLP44, PS3 and ACthio-1), which were found to display variable effects on the ER or the mitochondria-dependent pro-apoptotic axis. By contrast, FA4 activated pro-apoptotic pathways and decreased autophagy, except in MiaPaCa2 cells, in which autophagic proteins were expressed at lower levels and remained unmodified by FA4. FA4 treatment of PANC-1 xenografted mouse models, poorly responsive to conventional chemotherapy, significantly reduced tumor volumes and increased intratumor apoptosis compared to gemcitabine, with no signs of toxicity.

**Conclusions:**

Our data indicate that FA4 exhibits encouraging activity in pancreatic cancer cells unresponsive to gemcitabine. These results warrant further investigation in patient-derived pancreatic cancers, and hold promise for the development of therapies that can more efficiently target the specific characteristics of individual tumor types.

**Supplementary Information:**

The online version contains supplementary material available at 10.1007/s13402-021-00638-5.

## Introduction

**Fig. 1:**
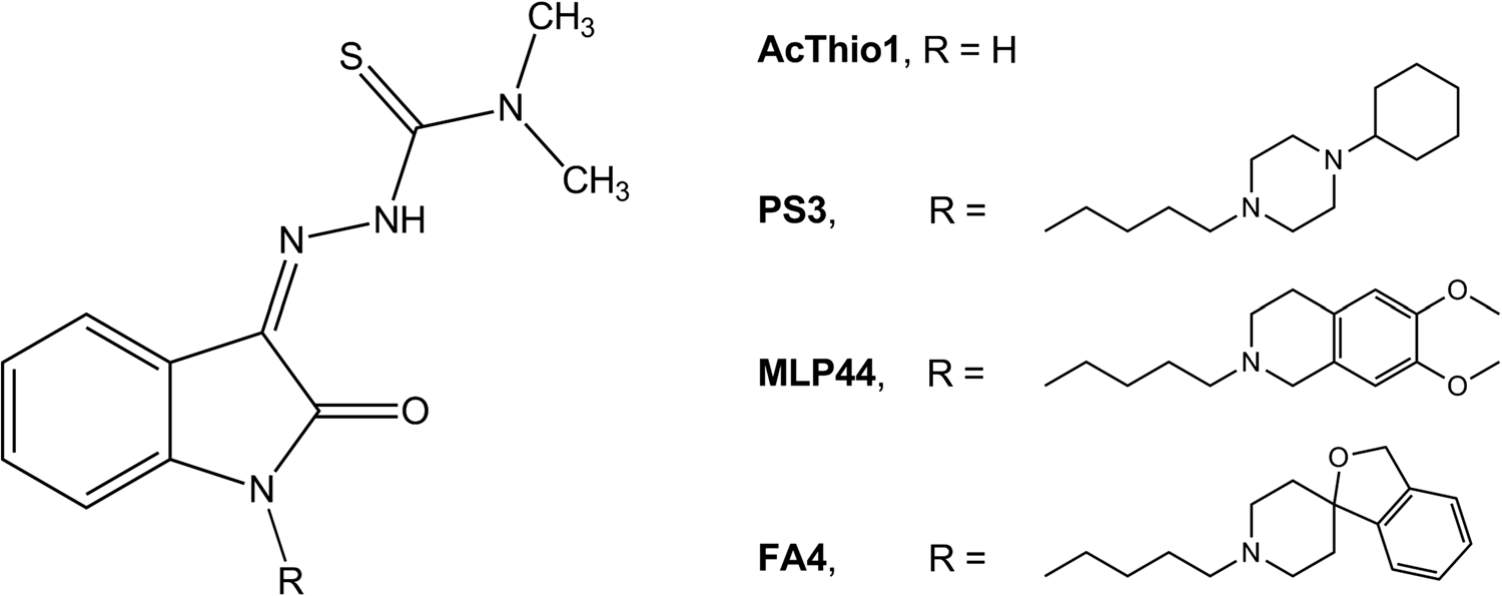
Structure of known (AcThio-1, PS3 and MLP44) and novel (FA4) thiosemicarbazones

Pancreatic cancer is one of the most aggressive forms of cancer characterized by a poor prognosis and a five-years survival rate of ~ 8 %. While the death rates have declined over the past two decades for the four major cancer types (lung, breast, prostate and colorectum), the death rate for pancreatic cancer has increased [1]. Surgery represents the first option when the disease is diagnosed early, with gemcitabine as a chemotherapeutic option towards which cancer cells may ultimately develop resistance. Therefore, there is an urgent need for alternative therapeutic strategies. With this aim, we previously produced a series of thiosemicarbazones that chelate metals and exhibit activity towards sigma-2 receptors and the drug efflux pump P-glycoprotein (P-gp) [2]. The strategy to simultaneously hit these targets in pancreatic cancer was based on (1) the observation that some sigma-2 receptor ligands are effective against pancreatic cancers [3–7] and (2) that cancer cells are sensitive to changes in energy levels because of their increasing energy need [8]. Interaction with the subtype 2 of sigma receptors, which are overexpressed in several cancers, activates different apoptotic pathways depending on the cell type and the molecule type involved [9]. Upon metal (iron and copper) chelation, thiosemicarbazones are able to alter the cell energy equilibrium. In an attempt to link these two activities, potent cytotoxic thiosemicarbazones that bind sigma-2 receptors have been obtained, and the impact on the synergistic action of the biological targets hit by these molecules (i.e., sigma-2 receptors, efflux pumps like P-gp and metal chelation) was studied using a deconstruction approach (Fig. 1). We evaluated the activity of these molecules in an *in vivo* preclinical model of pancreatic cancer and found that, while the multitarget strategy is not needed for the antitumor activity (the sole *N*,*N*-dimethylthiosemicarbazone chelating moiety is sufficient to confer cytotoxic action, as in compound ACthio-1, (*Z*)-*N,N*-dimethyl-2-(2-oxoindolin-3-ylidene)hydrazinecarbothioamide, Fig. 1), the presence of the sigma-2 targeting portion (as in compounds MLP44, (*Z*)-2-(1-(4-(6,7-dimethoxy-3,4-dihydroisoquinolin-2(1*H*)-yl)butyl)-2-oxoindolin-3-ylidene)-*N,N*-dimethylhydrazinecarbothioamide and PS3, (*Z*)-2-[1-[4-(4-cyclohexylpiperazin-1-yl)butyl]-2-oxoindolin-3-ylidene]-*N,N*-dimethylhydrazinecarbothioamide, Fig. 1) results in the activation of diverse cell death pathways and in a more specific delivery to tumors, leading to reduced off-target effects [4]. These promising results prompted us to produce a novel sigma-2 binding thiosemicarbazone, whose sigma-2 binding basic moiety was 3*H*-spiro[isobenzofuran-1,4’-piperidine]. The overall structure mimics the sigma-2 reference compound siramesine, which has been shown to be cytotoxic in a number of cells via pathways such as lysosomal leakage [10] and mitochondrial destabilization [11] that lead to oxidative stress [10–12]. We anticipated that insertion of such a moiety could result in an increased cytotoxic effect in pancreatic cancer cells by combining the potent action of the sigma-2 ligand siramesine with the metal chelating moiety of the thiosemicarbazone within one scaffold. The activity of this novel compound, named FA4, (*Z*)-2-(1-(4-(3* H*-spiro[isobenzofuran-1,4’-piperidine]-1’-yl)butyl)-2-oxoindolin-3-ylidene)-*N*,*N*-dimethylhydrazinecarbothioamide (Fig. 1), was tested in a panel of pancreatic cancer cell lines and challenged with our most promising thiosemicarbazones, either targeting sigma-2 receptors (MLP44 and PS3) or not (ACthio-1). While all the thiosemicarbazones studied were cytotoxic in the diverse pancreatic cancer cell lines tested, the type/presence of the basic moiety triggered different pathways in different cells, a result that looks promising in the perspective of a personalized medicine approach. Additionally, the novel compound performed better than the other thiosemicarbazones in all the pancreatic cancer cell lines tested, with a particular cytotoxic activity against the aggressive human PANC-1 cell line, which displays a reduced sensitivity to the first-line treatment agent gemcitabine [13]. The *in vitro* results were confirmed in PANC-1 xenografts, suggesting a potential of FA4 for the treatment of pancreatic cancers.

## Materials and methods

### Biological reagents and animals

[^3^H]-DTG (29 Ci/mmol) was purchased from PerkinElmer Life Sciences (Zavantem, Belgium), DTG was purchased from Tocris Cookson Ltd. (U.K.) and (+)-Pentazocine was obtained from Sigma-Aldrich-RBI s.r.l. (Milan, Italy). Wistar Hannover rats (250–300 g) were retrieved from Harlan, Italy. Cell culture reagents were purchased from EuroClone (Milan, Italy). MTT (3-[4,5-dimethylthiazol-2-yl]-2,5-diphenyltetrazoliumbromide) was obtained from Sigma-Aldrich (Milan, Italy). [10-(2,5-dihydroxy-3,4-dimethoxy-6,ethylphenyl)decyl]triphenyl-phosphonium, monomethanesulfonate (mitoquinol or mitoQ) was obtained from Cayman Chemical (Ann Arbor, MI, USA).

### Cell lines and culture conditions

Human pancreas cancer cell lines BxPC3 (CRL-1687™), AspC1 (CRL-1682™), MiaPaCa2 (CRL-1420™) and PANC-1 (CRL-1469™) were obtained from the American Type Culture Collection (ATCC®, Bethesda, MD, USA). The murine pancreas adenocarcinoma cell line PANC02 was a gift from Bryan Clary (Duke University). The KP02 mouse cell line was derived from pancreatic cancer tumor tissue obtained from p48-CRE/LSL-Kras^G12D^/p53^flox/+^ mice (backcrossed C57BL/6, n = 6). Non-tumor human pancreatic ductal epithelial (HPDE) cells were provided by Kerafast (cat. N° ECA001-FP, Boston, MA, USA). AspC1, BxPC3 and PANC02 cells were cultured in RPMI-1640 medium supplemented with 10 % fetal bovine serum (FBS). MiaPaCa2 cells were cultured in Dulbecco’s Modified Eagle’s Medium (DMEM) with 10 % FBS and 2.5 % horse serum. PANC-1 cells were cultured in DMEM with 10 % FBS. KP02 cells were cultured in a 1:1 mixture of DMEM and Ham’s F-12 Nutrient Mixture with 10 % FBS. HPDE cells were cultured in Keratinocyte/serum-free medium with EGF and bovine pituitary extract (Invitrogen). Penicillin (100 mg/ml) and streptomycin (100 mg/ml) were added to all media and the cells were maintained in a humidified incubator at 37 ^º^C with 5 % CO_2_.

### Flow cytometry

Flow cytometry studies to detect receptor densities and ligand binding affinities were carried out according to Abate et al. [14] and Niso et al. [15], for the sigma-1 and sigma-2 receptors, respectively. In order to detect the sigma-2 receptor content together with the affinity of FA4 for sigma-2 receptors in the same cells, MiaPaCa2, PANC-1, BxPC3, PANC02, KP02, AspC1 and HPDE cells were incubated with increasing concentrations (1, 10 and 100 nmol/L and 1 and 10 µM) of FA4, followed by 100 nmol/L of sigma-2 fluorescent compound (NO1, 2-{6-[2-(3-(6,7-dimethoxy-3,4-dihydroisoquinolin-2(1* H*)-yl)propyl)-3,4-dihydroisoquinolin-1(2* H*)-one-5-yloxy]hexyl}-5-(dimethylamino)isoindoline-1,3-dione) [15] for 75 min at 37 °C. To mask sigma-1 receptors, (+)-pentazocine (10 µM) was co-incubated. As a validation procedure, the same experiment was repeated with the sigma-2 reference compound DTG instead of FA4. At the end of the incubation periods, the cells were washed twice with PBS, detached with 200 ml Cell Dissociation Solution (Sigma Chemical Co.) for 10 min at 37 °C, centrifuged at 13,000 g for 5 min and resuspended in 500 µl PBS. Fluorescence was recorded using a Bio-Guava® easyCyte™ 5 Flow Cytometry System (Millipore, Billerica, MA, USA) equipped with a 530 nm band pass filter. For each analysis, 50,000 events were collected and analyzed using InCyte software (Millipore).

The sigma-1 receptor density and FA4 binding to sigma-1 receptors was measured in PANC-1, MiaPaCa2 and HPDE cells that were incubated with increasing concentrations (1, 10 and 100 nmol/L and 1 and 10 µM) of (+)-pentazocine or FA4, followed by 100 nmol/L sigma-1 fluorescent compound (LM1, 5-(dimethylamino)-2-(6-((5-(4-(4-methylpiperidin-1-yl)butyl)-5,6,7,8-tetrahydronaphthalen-2-yl)oxy)hexyl)isoindoline-1,3-dione) [14] for 75 min at 37 °C. To mask sigma-2 receptors, the sigma-2 receptor selective ligand F390, 2-(3-(6,7-dimethoxy-3,4-dihydroisoquinolin-2(1* H*)-yl)propyl)-5-methoxy-3,4-dihydroisoquinolin-1(2* H*)-one [16] (10 µM) was co-incubated. At the end of the incubation periods, the cells were washed twice with PBS, detached with 200 ml Cell Dissociation Solution (Sigma Chemical Co.) for 10 min at 37 °C, centrifuged at 13,000 g for 5 min and resuspended in 500 µl PBS. Fluorescence was recorded using a Bio-Guava® easyCyte™ 5 Flow Cytometry System (Millipore, Billerica, MA, USA), equipped with a 530 nm band pass filter. For each analysis, 50,000 events were collected and analyzed using InCyte software (Millipore).

### Sigma-2 binding assay

Sigma-2 receptor binding was carried out according to Berardi et al. [17]. [^3^H]-DTG was used as sigma-2 receptor-specific radioligand in the presence of 1 µM (+)-pentazocine to mask sigma-1 receptors, in rat liver membranes. DTG (85–96 %) was used for specific binding calculation. Concentrations required to inhibit 50 % of radioligand-specific binding (IC_50_) were determined using six to nine different concentrations of the drug in two or three experiments with samples in duplicate. Scatchard parameters (*K*_d_ and *B*_max_) and apparent inhibition constant (*K*_i_) values were determined by nonlinear curve fitting, using Prism version 5.0, GraphPad software.

### Cell viability assay

Cell viability was assessed using a MTT assay at 48 h [3, 18]. On day 1, 25,000 cells/well were seeded into 96-well plates in 100 µl culture medium. On day 2, drugs at concentrations ranging from 1 µM to 100 µM were added. In all experiments the drug-solvents (EtOH, DMSO) were added as controls to evaluate possible solvent cyto-toxicities. After the established incubation time with drugs (48 h), MTT (0.5 mg/ml) was added to each well, and after 3–4 h incubation at 37 °C, the supernatants were removed. The formazan crystals formed were solubilized using 100 µl DMSO/EtOH (1:1) after which the absorbance values at 570 and 630 nm were determined using a microplate reader Victor 3 from PerkinElmer Life Sciences.

### Caspase 3, 7 and 9 activity assays

Caspase 3, caspase 7 and caspase 9 activation was measured using a Caspase 3/7 Fluorescence Assay kit (Cayman Chemical, Ann Arbor, MI, USA) and a Caspase 9 fluorometric assay kit **(**Enzo Life Science, Roma, Italy). The results are expressed as nmol of the hydrolyzed substrate of each caspase/mg cellular proteins, according to a previously set titration curve.

### qRT-PCR assay

Total RNA was extracted using a phenol/chloroform method, after which 1 µg RNA was reverse-transcribed using an iScript Reverse Transcription Supermix kit according to the manufacturer’s instructions (Bio-Rad Laboratories). 25 ng cDNA was amplified using 10 µl of an IQ™ SYBR Green Supermix (Bio-Rad Laboratories). Primers were designed using qPrimer Depot software (http://primerdepot.nci.nih.gov/). qRT-PCR was carried out using an iQ^TM^5 cycler (Bio-Rad Laboratories). The cycling conditions used were: 30 s at 95 °C, followed by 40 cycles of denaturation (15 s at 95 °C) and annealing/extension (30 s at 60 °C). The same cDNA preparations were used to quantify the expression of genes of interest and that of the housekeeping gene *S14*, which was used to normalize the gene expression levels. Relative quantitation of each sample was performed using Gene Expression Quantitation software (Bio-Rad Laboratories). The results were expressed in arbitrary units. For each gene, the expression level in untreated cells was arbitrarily set at “1”.

### Mitochondrial and total ROS measurements

Intra-mitochondrial ROS was measured using fluorescent probe MitoSOX as per manufacturer’s instructions (Invitrogen). To measure total ROS, cells were incubated with the ROS-sensitive probe 5-(and-6)-chlorometyl-20,70-dichlorodihydro-fluorescein diacetate-acetoxymethyl ester (5 mmol/L; DCFDA-AM) as described by Riganti et al. [19]. The results are expressed as nmol/mg mitochondrial or cellular protein, respectively.

### Mitochondrial depolarization assay

Staining with JC-1 fluorescent probe (Biotium Inc., Freemont, CA, USA) was performed as detailed in Riganti et al. [19]. The resulting fluorescence units were used to calculate the percentage of green-fluorescent (i.e., depolarized) mitochondria versus red-fluorescent (i.e., polarized) mitochondria.

### Western blotting

Cells were rinsed with ice-cold lysis buffer (50 mM, Tris, 10 mM EDTA, 1 % v/v Triton-X100), supplemented with protease inhibitor cocktail set III (80 µM aprotinin, 5 mM bestatin, 1.5 mM leupeptin, 1 mM pepstatin; Calbiochem, San Diego, CA, USA), 2 mM phenylmethylsulfonyl fluoride and 1 mM Na_3_VO_4_, then sonicated and centrifuged at 13,000 g for 10 min at 4 ^o^C. 20 µg protein extracts were subjected to SDS-PAGE and probed with antibodies directed against ATG5, ATG7, ATG12, beclin, p62, LC3, GRP78, ATF6, IRE-1α and PERK (all from Abcam, Cambridge, UK), followed by incubation with a peroxidase-conjugated secondary antibody (Bio-Rad Laboratories). Next, the membranes were washed with Tris-buffered saline-Tween 0.1 % v/v solution, and proteins were detected by enhanced chemiluminescence (Bio-Rad Laboratories). To check for equal loading, the samples were probed with an anti-β-tubulin antibody (Santa Cruz Biotechnology Inc., Santa Cruz, CA, USA).

### Pro-oxidant and anti-oxidant enzyme activity assays

The activity of NADPH oxidase was measured in cell lysates using a chemiluminescence-based assay reported by Tassone et al. [20]. The results were expressed as RLU/mg cellular protein. Superoxide dismutase and catalase activities were measured using a colorimetric Superoxide Dismutase Activity Assay kit (Abcam) and a Catalase Activity Assay kit (Abcam), respectively, as per manufacturer’s instructions. The results were expressed as optical density (OD)/mg cellular protein.

### ***In vivo*** xenograft assays

2 × 10^5^ PANC-1 or Mia-PaCa2 cells were inoculated subcutaneously in the right flank of 6-week old C57BL/6 female nude mice (Charles River Laboratories Italia, Calco), housed (5 per cage) under a 12 h light/dark cycle, with food and drinking water provided *ad libitum*. Tumor growth was measured daily by caliper, according to the equation (LxW^2^)/2, where L = tumor length and W = tumor width. When tumors reached a volume of 100 mm^3^, the mice (*n* = 8/group) were randomized in the following groups and treated daily for 15 days or 30 days intraperitoneally as reported: (1) Vehicle group (100 µl saline solution), (2) FA4^*low*^ group (750 nmol FA4 in 100 µl saline solution), (3) FA4^*high*^ group (1500 nmol FA4 in 100 µl saline solution) and (4) Gemcitabine group (20 mg/kg gemcitabine, twice a week). Tumor volumes and animal weights were monitored daily. Animals were euthanized at day 18 or 36 after randomization with zolazepam (0.2 ml/kg) and xylazine (16 mg/kg). Next, tumors were excised and paraffin-embedded. Sections were immuno-stained for cleaved caspase 3 (Cell Signalling Technology, Danvers, MA, USA) or with an anti-malondialdehyde antibody (Abcam), followed by a peroxidase-conjugated secondary antibody (Dako, Glostrup, Denmark). The sections were examined under a Leica DC100 microscope (Leica, Wetzlar, Germany). Quantification of the immunohistochemical analyses was performed using ImageJ software (https://imagej.nih.gov/). The staining intensities were expressed as arbitrary units and considered 1 in the vehicle group. Red blood cell (RBC) count, hemoglobin (Hb), white blood cell (WBC) count, platelet (PLT) count, lactate dehydrogenase (LDH), aspartate aminotransferase (AST), alanine aminotransferase (ALT), alkaline phosphatase (AP), creatinine and creatine phosphokinase (CPK) were measured in blood samples collected immediately after euthanasia, using commercially available kits from Beckman Coulter Inc (Beckman Coulter, Miami, FL, USA). In all cases, researchers analyzing the results were unaware of the treatments received by the animals. The study complied with the ARRIVE guidelines and was approved by the Bio-Ethical Committee of the Italian Ministry of Health (#122/2015-PR).

### Statistical analysis

Unless specified otherwise, data plotting and statistical analysis were conducted using GraphPad Prism 5.0. Data were analyzed by applying the one-way repeated measures analysis of variance, and Bonferroni’s multiple comparison test followed as a post hoc test. The results are reported as mean ± SEM (standard error of the mean) of at least two to three independent experiments, performed in triplicate. Statistical significance was accepted at *p* < 0.05.

## Results

### FA4 synthesis

According to a previously developed procedure, FA4 was synthesized starting from alkylation of 3*H*-spiro[isobenzofuran-1,4’-piperidine] [21] with 1-(4-chlorobutyl)indoline-2,3-dione 1 in the presence of K_2_CO_3_, [2] providing the amine 2. This intermediate amine was transformed into its corresponding hydrochloride salt, dissolved in hot EtOH and treated with 4,4-dimethyl-3-thiosemicarbazide to afford final thiosemicarbazone FA4 as hydrochloride salt (Scheme S1, Supplementary Information). The experimental synthetic procedures are provided in the Supplementary Information.

### Affinity of FA4 at sigma-2 receptors

The binding of FA4 at sigma-2 receptors, as measured by a classical radioligand binding assay, was found to be notable (*K*_i_ = 15.8 nM, Table 1) and in strict agreement with the binding affinity of the siramesine lead compound (*K*_i_ = 12.6 nM) [8], indicating that the thiosemicarbazone moiety was not detrimental for sigma-2 receptor binding.

**Table 1:** Binding affinity values of FA4 and reference compounds at sigma receptors

	Binding assay						
Compound	Radioligand, *K*_i_ ± SEM^a^ (nΜ)	Flow Cytometry, IC_50_ ± SEM^a^ (µM)					
	*Sigma-2*	*Sigma-2*				*Sigma-1*	
	Rat Liver	MiaPaCa2	PANC-1	AspC1	KP02	MiaPaCa2	PANC-1
FA4	15.8 ± 3.6	9.13 ± 1.1	11.4 ± 2.3	10.6 ± 2.2	11.6 ± 1.5	53.2 ± 5.6	51.3 ± 4.3
DTG	22.5 ± 3.6	5.98 ± 0.9	6.59 ± 1.6	14.3 ± 3.0	7.16 ± 1.2		
(+)-pentazocine						12.8 ± 2.1	29.3 ± 3.9

^a^ Values represent the mean of ≥ 3 separate experiments in duplicate ± SEM

### Density of sigma-2 receptors and binding affinity of FA4 in normal (immortalized) and pancreatic cancer cells

The presence of sigma-2 receptors was assessed in a panel of pancreatic cells. To this end, flow cytometry was conducted in human (MiaPaCa2, PANC-1, AsPC1 and BxPC3) and murine (KP02, PANC02) pancreatic cancer cells, as well as in HPDE cells. The assay was performed by incubating each cell line with increasing concentrations of the selective sigma-2 fluorescent ligand NO1 [15, 22]. Saturation of the sigma-2 receptors in each cell line and assessment of non-specific binding through displacement with DTG allowed us to define specific binding. The results indicated that sigma-2 receptors were 1.8- to 3.2-fold more abundantly expressed in pancreatic cancer cells than in HPDE cells (Fig. S1 – A, Supplementary Information). The only exception was the KP02 cell line in which the density of sigma-2 receptors was comparable to that in HPDE cells. Using flow cytometry, we measured the binding affinity of FA4 at sigma-2 receptor subtypes in the pancreatic cancer cells according to previously developed procedures [15]. Binding curves were generated for the thiosemicarbazone upon dose-dependent displacement of the fluorescent ligand NO1 [15] with FA4 leading to IC_50_ values that line up with the results from the radioligand binding assay (IC_50_ values ranging from 9.13 nM to 11.6 nM in MiaPaCa2, PANC-1, Aspc1 and KP02 cells, Table 1), showing an equally high nanomolar affinity in these representative cell lines.

### Density of sigma-1 receptors and binding affinity of FA4 in normal (immortalized) and pancreatic cancer cells

Since we found that siramesine binds equally well to the sigma-1 and sigma-2 receptor subtypes [8], we next investigated the presence of sigma-1 receptors together with the binding of FA4 for this subtype in clinically relevant pancreatic cancer cells (PANC-1 and MiaPaCa2). The fluorescent ligand LM1 [14] was used to measure the sigma-1 receptor density in the above cells and HPDE cells upon masking of the sigma-2 subtype with the selective ligand F390 [16] (Fig. S1 – B, Supplementary Information). The presence of the sigma-1 receptor was ascertained, with no apparent differences in density between the tumor and immortalized cells, in contrast to the sigma-2 receptor. Additionally, the binding curves generated upon dose-dependent displacement of LM1 in PANC-1 and Miapaca-2 cells indicated a moderate affinity of FA4 for sigma-1 receptors (IC_50_ values = 51.3 and 53.2 nM, respectively, Table 1), suggesting a more pronounced sigma-2 than sigma-1 mediated action of FA4 in these cells.

### Cytotoxic activity of FA4 in normal (immortalized) and pancreatic cancer cells

The cytotoxic activity of the novel thiosemicarbazone FA4 was evaluated in human (MiaPaCa2, PANC-1, AsPC1, BxPC3) and murine (KP02, PANC02) pancreatic cancer cells harboring diverse driver mutations (Table 2). We found that FA4 showed relevant low micromolar cytotoxic activities in all the cell lines studied (EC_50_ ranging from 0.88 µM to 3.01 µM, Table 2). In particular, in PANC-1, MiaPaCa2 and KP02 cells, the FA4 activity was 3- to 8-fold higher compared to that conferred by the other sigma-2 targeting thiosemicarbazones MLP44 and PS3. The cytotoxicity of all thiosemicarbazones was also measured in HPDE cells in which the compounds generally showed a less potent activity than in the cancer cells, but again, only FA4 consistently exhibited a 2- to 7- fold lower cytotoxicity (EC_50_ = 6.11, Table 2).

**Table 2:** Activity values of thiosemicarbazones compounds in pancreatic cell lines

Cmpd	Activity in pancreatic cell lines, EC_50 _± SEM^a^ (µM)						
	Cancer cells						Normal cells
	MiaPaCa2	PANC-1	AspC1	BxPC3	KP02	PANC02	HPDE
FA4	1.98 ± 0.4	3.01 ± 0.8	1.39 ± 0.3	1.36 ± 0.2	0.88 ± 0.1	1.94 ± 0.5	6.11 ± 1.1
MLP44^b^	14.6	14.6	2.01	2.34	6.92	1.33	9.50
PS3^b^	10.08	8.73	3.86	6.15	7.32	1.17	5.35
Acthio1^b^	18.3	> 100	2.17	2.52	2.83	1.21	10.5

^a^Values represent the mean of ≥ 3 separate experiments in duplicate ± SEM; ^b^From ref [4]

### FA4 induces pancreatic cancer cell apoptosis by eliciting endoplasmic reticulum stress and mitochondrial damage

**Fig. 2:**
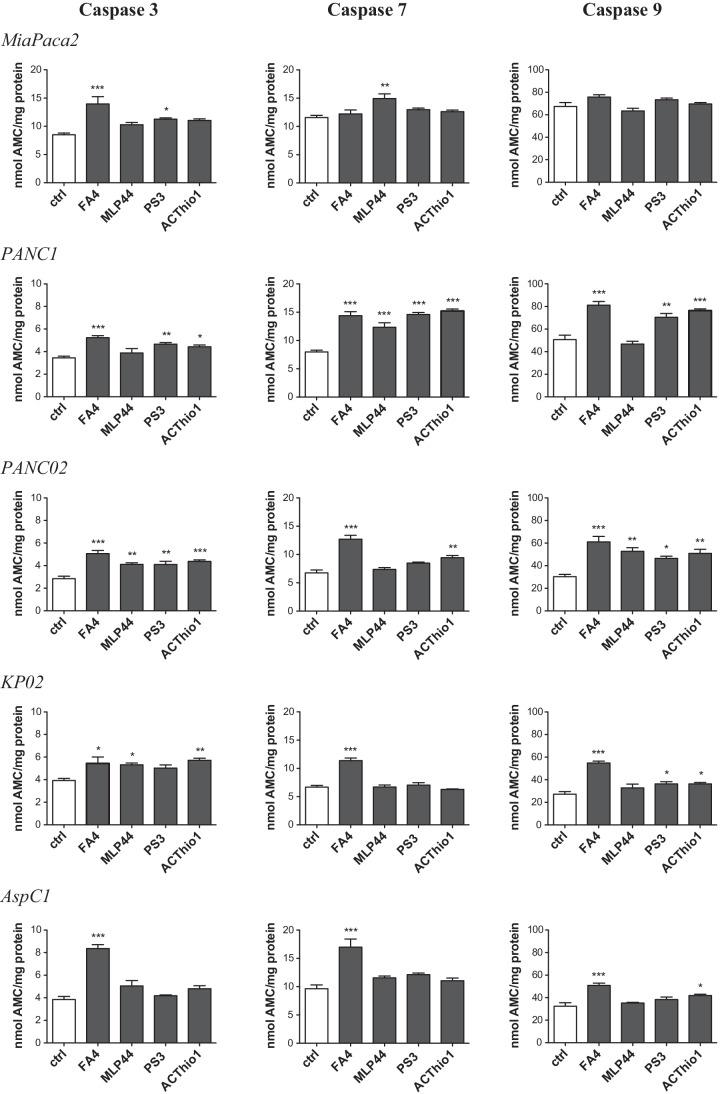
Activation of caspase 3, 7 and 9 by thiosemicarbazones in pancreatic cancer cells. Fluorimetric measure of caspase 3, 7 and 9 in cells treated 2 h with 50 µM of each compound. Results are shown means ± SEM (*n* = 3), * *p* < 0.05

Next, we investigated whether the different compounds induce pancreatic cancer cell apoptosis, an effect that has already been reported for sigma-2 receptor ligands in other cancer cells [3, 23, 24]. We first measured the activity of caspase 3, i.e., the caspase that irreversibly determines apoptotic death, in human (MiaPaCa2, PANC-1, AspC1) and murine (KP02, PANC02) cells, treated with the sigma-2 targeting thiosemicarbazones FA4, MLP44, PS3 and the metal chelator ACthio-1 (Fig. 2), using the same experimental conditions (50 µM for 2 h) under which sigma-2 receptor ligands have previously been found to induce cytotoxic effects against pancreatic cancer cells [3]. FA4 activated caspase 3 in all the cell lines investigated, while the other compounds activated caspase 3 in a variable and cell-dependent manner. The latter thiosemicarbazones did, for instance, not activate caspase 3 in MiaPaCa2 and AspC1 cells, but activated caspase 3 in PANC02 and KP02 cells, in partial agreement with previous findings [4]. Moreover, FA4 was the most potent inducer of caspase 3 in all cell lines tested compared to the other compounds (with the only exception of ACthio-1 having a similar activity in KP02 cells). Notably, FA4 was a significant inducer of caspases in all the cell lines analyzed, except for MiaPaCa2, also when used at a concentration corresponding to its IC50 (Supplementary Fig. S2).

**Fig. 3:**
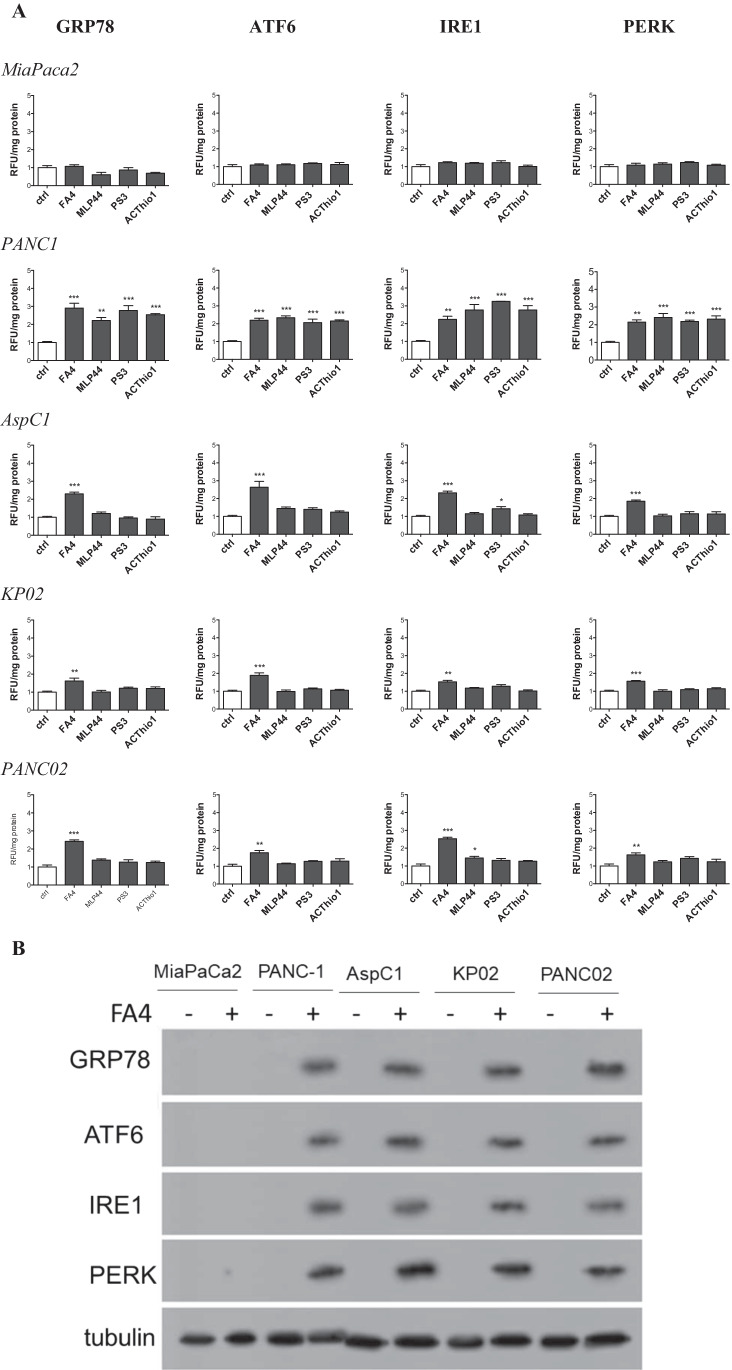
ER markers in thiosemicarbazone-treated pancreatic cancer cells. **(A)** mRNA expression of ER stress markers, measured by qRT-PCR, in cells treated 2 h with 50 µM of each compound. Results are means ± SEM (n = 3), * *p* < 0.05. **(B)** Western blot analysis of GRP78, ATF6, IRE1 and PERK in the indicated cell lines incubated with FA4 at 50 µM for 2 h. The image is representative of three independent experiments. Tubulin was used as control for equal protein loading

To better understand the biochemical mechanisms of FA4 and the other compounds in inducing apoptosis, we first focused on their effects exerted on the endoplasmic reticulum (ER), an intracellular compartment where sigma-2 receptors have been reported to reside [25]. Sigma-2 receptors are known inducers of ER stress, a condition that perturbs the correct folding of proteins within the ER and causes the so-called unfolded protein response (UPR). UPR is sensed by the chaperone glucose-regulated protein 78 (GRP78) and by sensors activating factor 6 (ATF6), i.e., inositol-requiring enzyme-1α (IRE-1α) and PKR-like ER kinase (PERK), that activate downstream effectors leading to cell survival if the ER stress is short and reversible, or to cell apoptosis by activating the caspase 7/caspase 3 axis if the stress persists [26]. By altering calcium flux, sigma-2 receptor modulators are known to induce ER stress [27] and to promote cell death by activating apoptotic and autophagic pathways [28]. The expression of ER stress markers such as GRP78, ATF6, IRE1 and PERK was evaluated in all the pancreatic cancer cells (Fig. 3A). The four ER stress markers increased upon treatment with FA4 in all the cells tested, except in MiaPaCa2 cells. All the thiosemicarbazones increased the expression of these markers in PANC-1, whereas none of the markers was increased in the other cells by MLP44, PS3 and ACthio-1. The increase in ER stress induced by FA4 was validated by Western blotting (GRP78, ATF6, IRE1 and PERK; Fig. 3B). The results obtained confirmed that in all cell lines except MiaPaCa2, this thiosemicarbazone was able to increase the expression of ER stress sensors and executers. Notably, FA4 increased the expression of ER stress markers also when used at its IC50 concentration (Supplementary Fig. S3). In agreement with the ER stress marker expression modulation, caspase 7, which is activated upon ER stress, was activated by FA4 in all cell lines except MiaPaCa2 (Fig. 2, Supplementary Fig. S2). Similarly, we found that all the thiosemicarbazones increased caspase 7 in PANC-1 cells, while the effect in the other cell lines was highly variable. The activation of caspase 7 may be responsible for downstream activation of caspase 3, although the levels of caspase 7 and caspase 3 activation are not always comparable in the same cell lines treated with the same compound. These small discrepancies may be due to different pools of pro-apoptotic and anti-apoptotic factors present in each cell line which may be affected differently by the compounds. Alternatively, other mechanisms converging on the activation of caspase 3 could be envisioned.

**Fig. 4:**
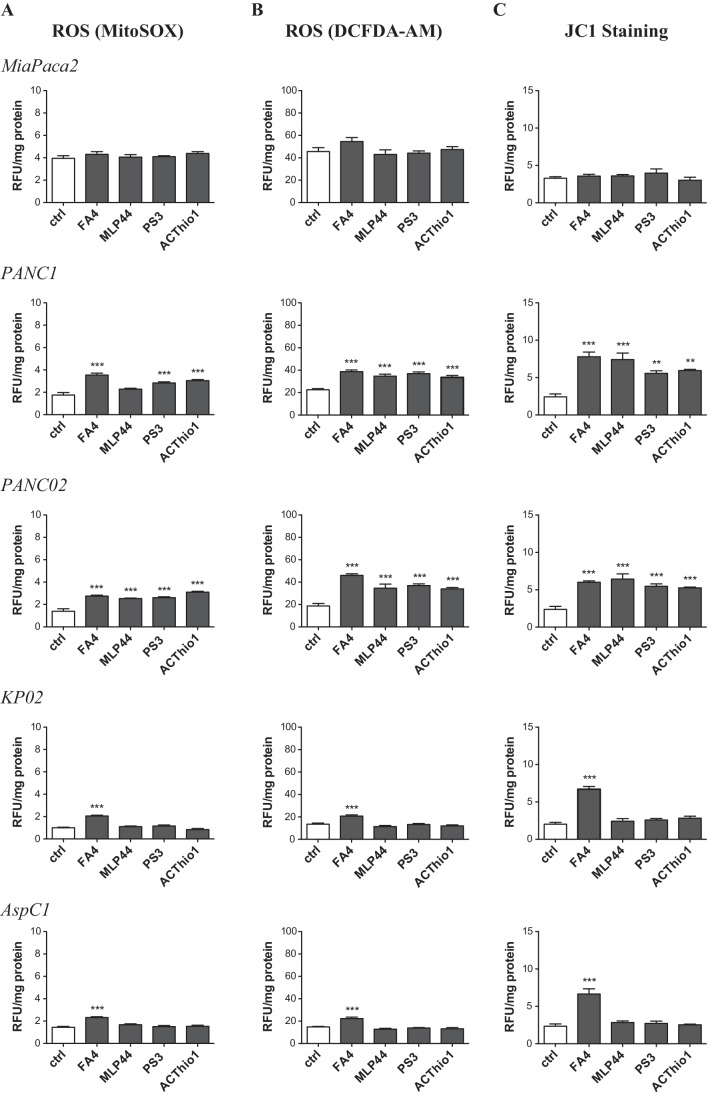
ROS and mitochondrial damage markers in thiosemicarbazones-treated pancreatic cell lines. Fluorometric staining of mitochondrial ROS (MitoSOX staining, (**A**), whole cell ROS (DCFDA-AM probe, (**B**) and mitochondrial depolarization (JC1 staining, (**C**) in cells treated 2 h with 50 µM of each compound. Results are means ± SEM (*n* = 3), * *p* < 0.05

Alterations in calcium homeostasis in the so-called mitochondria-associated ER membranes (MAM) may also alter its metabolic functions, leading to calcium overload, increased production of reactive oxygen species (ROS), mitochondrial depolarization followed by activation of cell death signals triggered by the caspase 9/caspase 3 axis and autophagy [29]. Importantly, sigma receptors have been found to reside in the MAM cell compartments, where in particular the sigma-1 subtype regulates Ca^2+^ fluxes between the ER and mitochondria [30]. We thus focused on mitochondria-related events as possible triggers of additional pro-apoptotic mechanisms. Interestingly, FA4 increased mitochondrial ROS in all the cell lines tested, except MiaPaCa2, whereas the other thiosemicarbazones increased mitochondrial ROS only in PANC-1 and PANC02 cells (Fig. 4). The levels of ROS in whole cells (Fig. 4) reflected the levels of mitochondrial ROS, suggesting that ROS can diffuse from mitochondria to the cytosol. Alternatively, we may speculate that thiosemicarbazones are able to increase ROS also in a mitochondria-independent way, e.g. by increasing cytosolic ROS-producing enzymes such as NADPH oxidase or reducing the activity of anti-oxidant enzymes, such as superoxide dismutase 1, catalase, peroxidases, thioredoxins. Preliminary data on the activity of NADPH oxidase, superoxide dismutase 1 and catalase (Supplementary Fig. S4), however, seem to exclude this latter possibility.

**Fig. 5:**
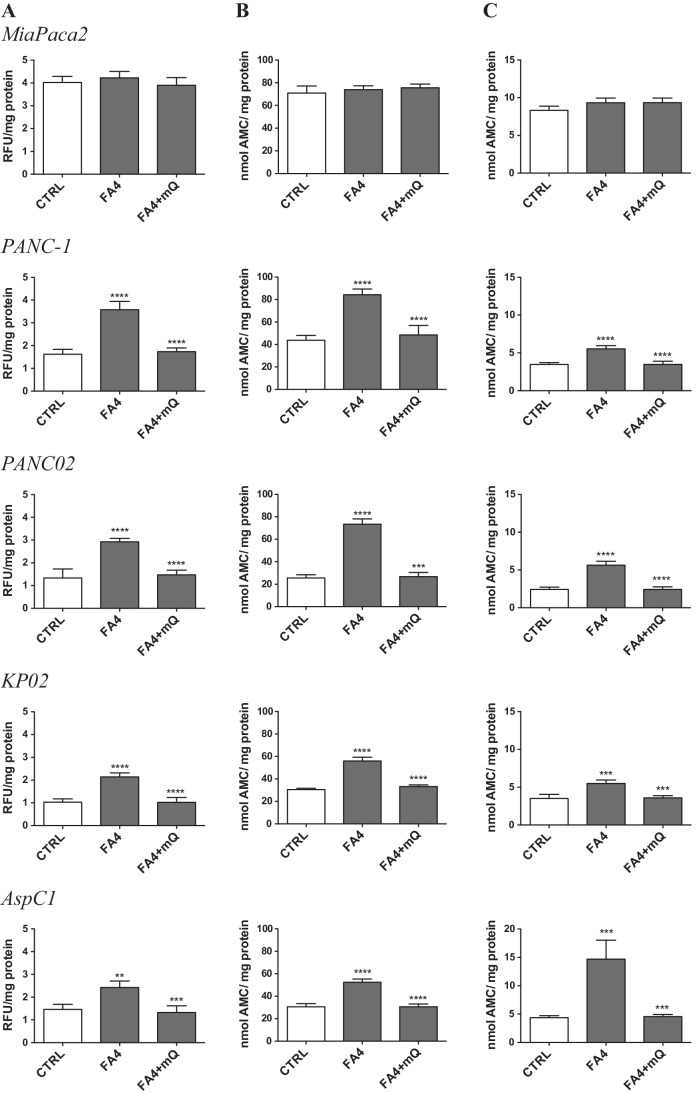
Mitochondrial ROS (**A**), activation of caspase 9 (**B**) and caspase 3 (**C**) in cancer cells treated 2 h with 50 µM of FA4 alone or plus 0.4 µM mitoquinol, chosen as scavenger of mitochondrial ROS. Results are means ± SEM (*n* = 3), * *p* < 0.05

An increase in mitochondrial ROS may be harmful to cancer cells. Indeed, staining with JC1, a dye sensitive to mitochondrial depolarization, indicated that all FA4-treated cells had depolarized (i.e., damaged) mitochondria, except MiaPaCa2 (Fig. 4). The other thiosemicarbazones increased mitochondrial depolarization only in PANC-1 and PANC02 cells, but not in the other cell lines tested (Fig. 4). This parallelism between mitochondrial ROS and depolarization suggests that the latter event is a consequence of increased mitochondrial ROS. Mitochondrial damage triggers the activation of caspase 9, and this was observed in all the cells in which FA4 increased mitochondrial ROS and depolarization (Figs. 2 and 4). By contrast, we found that the other compounds had a variable effect on the activation of caspase 9, dependent on the cell line and not strictly correlated with mitochondrial ROS and depolarization. Again, MiaPaCa2 cells were completely refractory to the activation of caspase 9 (Fig. 2). As proof of concept that mitochondrial ROS triggers cell death induced by FA4, we treated the cells with the mitochondrial ROS scavenger mitoquinol (mitoQ), at a concentration that abrogates the increase in mitochondrial ROS elicited by FA4 (Fig. 5A). Under these experimental conditions we did not find any activation of caspase 9 and caspase 3 in the FA4-treated cells (Fig. 5B-C).

**Fig. 6:**
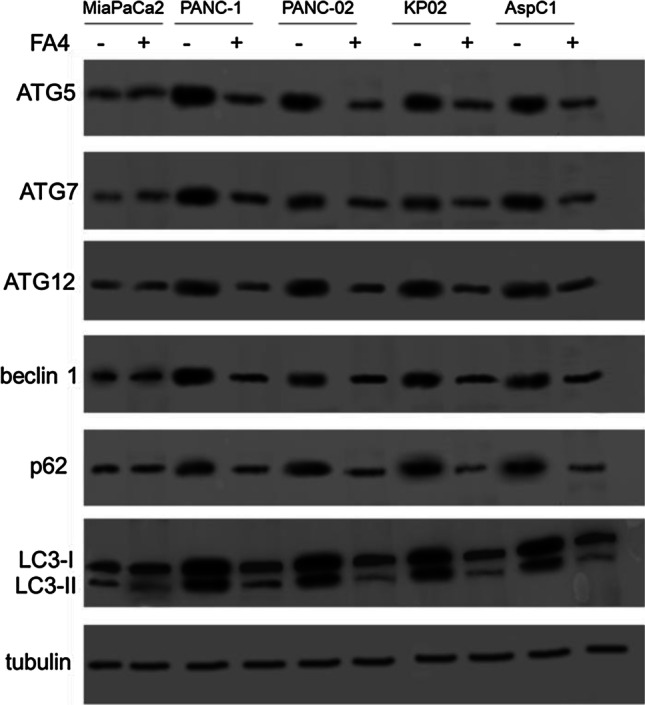
Expression of authophagy and protein sequestration markers in pancreatic cancer cell lines. Western blot of the indicated proteins in cells treated 2 h with 50 µM of FA4. The image is representative of three independent experiments. Tubulin was used as control for equal protein loading

To explain the absence of activation of ER stress- and mitochondrial stress-dependent pro-apoptotic pathways in MiaPaCa2 cells, in contrast to the other cell lines, we assessed whether cells differ in autophagy that contributes to the apoptosis induced by sigma-2 receptors following ER [28] or mitochondrial [29] stress. Interestingly, we found that all responsive cell lines exhibited higher levels of autophagosome proteins (ATG5, ATG7, ATG12, beclin 1), sequestration markers (p62) and LC3-II/LC3-I ratios than MiaPaCa2 cells. Moreover, FA4 reduced the levels of all the above-mentioned proteins as well as the LC-I/LC-II conversion in responsive cells, but not in MiaPaCa2 cells (Fig. 6).

### *In vivo* antitumor activity of FA4

The potent *in vitro* antitumor activity shown by FA4 in the aggressive human PANC-1 cells prompted us to investigate whether the anticancer effect is translated *in vivo* as well. In a first experiment, mice carrying PANC-1 and MiaPaCa2 xenografts were treated for 15 days with two dosages (^*low*^ and ^*high*^) of FA4, following the protocol adopted for other thiosemicarbazones [4]. Gemcitabine was used for comparison, because it is a standard treatment in pancreatic cancer. We found that at the end of the treatment periods the tumors were significantly smaller in mice treated with FA4 750 ^*low*^ and FA4 1500 ^*high*^ compared to vehicle in the PANC-1 xenografts. An important reduction in tumor volume for both FA4 regimens was also recorded in PANC-1 tumors in comparison with mice treated twice weekly with gemcitabine, which was ineffective against this tumor cell line (Fig. S5A, Supplementary Information). By contrast, we found that MiaPaCa2 tumors were more sensitive to gemcitabine, but in line with the data observed *in vitro*, FA4 was ineffective at both dosages (Fig. S5B, Supplementary Information).

**Fig. 7:**
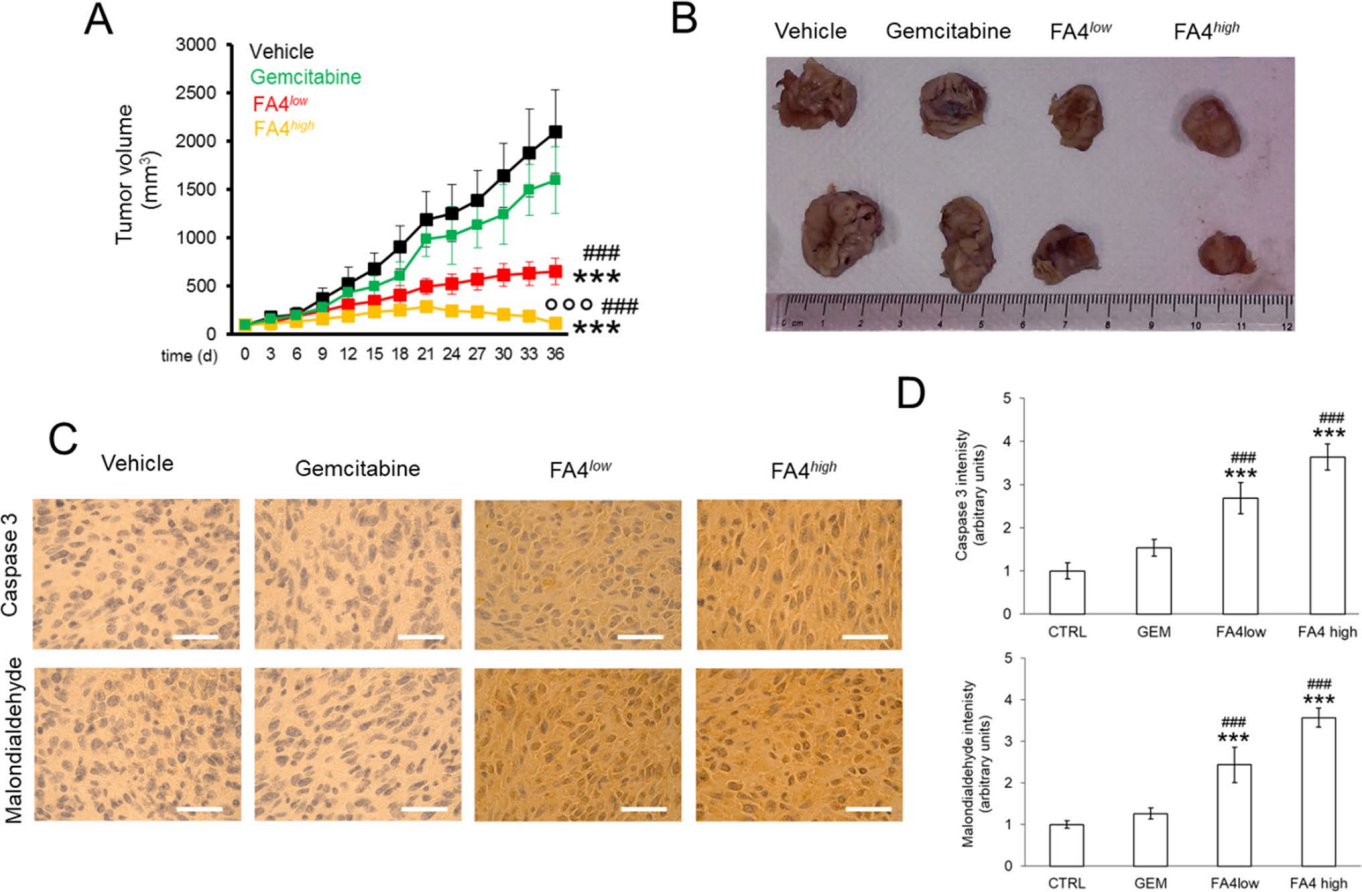
FA4 in C57BL/6 mice bearing PANC-1 tumors, treated for 30 days. (1) Vehicle group (black line, 100 µl saline solution); (2) FA4^*low*^ group (red line, 750 nmoles FA4 in 100 µl saline solution); (3) FA4^*high*^ group (yellow line, 1500 nmoles FA4 in 100 µl saline solution); (4) Gemcitabine group (green line, 20 mg/kg gemcitabine, twice a week). Animals were euthanized at day 36. **(A)** Tumor growth. Results are means ± SEM (n = 8). *** *p* < 0.001: FA4-groups vs. vehicle (days 21–36); ^##^ *p* < 0.001: FA4-groups vs. gemcitabine (days 21–36); °°° *p* < 0.001: FA4^*high*^-group vs. FA4^*low*^-group (days 21–36). **(B)** Representative photos of excised tumors. **(C)** Immunohistochemical analysis of intratumor cleaved caspase 3 and malondialdehyde, as index of lipid peroxidation. The images are representative of each group of treatment. Ocular: 10x; objective: 20x. Bars: 50 μm. **(D)** Quantification of the immunohistochemical staining performed using Image J software. Results are means ± SEM (n = 8). *** *p* < 0.001: FA4-groups vs. vehicle; ^##^ *p* < 0.01, ^###^P < 0.001: FA4-groups vs. gemcitabine

In a second experimental set-up, we prolonged the treatment of PANC-1 tumor-bearing mice for 30 days, to evaluate the effects in terms of tumor growth rate and systemic toxicity. Under these conditions, while gemcitabine was not able to significantly reduce tumor growth, FA4 at the low dosage showed a cytostatic effect and at higher dosages regression of tumor growth (Fig. 7A-B). Moreover, in line with the increased ROS and apoptosis observed in PANC-1 cells, intratumor apoptosis, measured as cleaved caspase 3 positivity, and lipid peroxidation, considered an index of intratumor oxidative stress, were low or undetectable in untreated and gemcitabine-treated animals, as well as in animals treated with low dosages of FA4, but became more pronounced in tumors from animals treated with high dosages of FA4 (Fig. 7C-D). No treatment-related deaths, weight loss or abnormalities in mouse behavior were observed. Also, no significant differences were measured in blood cell counts and hemato-chemical parameters (AST, ALT, LDH, CPK, creatinine), and no significant differences were noted between FA4 treated mice (at both the concentrations used) and when compared to the control group (Table S1, Supplementary Information).

## Discussion

Multifunctional thiosemicarbazones that bind sigma receptors and chelate metals have provided promising results in pancreatic cancer models both *in vitro* and *in vivo*. Here, a panel of human and murine genotypically and phenotypically different pancreatic cancer cells [31] was selected, since previous experiments have shown that different pancreatic cancer cells may respond differently to chemotherapeutic agents [4]. In all the cell lines included, the presence of sigma-2 receptors was evaluated by flow cytometry. We found that the expression of the sigma-2 receptor in the cancer cells was 2 to 3 –fold higher compared to that in non-tumor epithelial cells. On the other hand, we found that the sigma-1 receptor subtype was equally expressed in human pancreatic cancer cells and in the non-tumor immortalized counterparts. The binding affinity of the novel thiosemicarbazone FA4 for sigma-2 receptors was evaluated using a classical radioligand binding assay, revealing a low nanomolar *K*_i_ value consistent with the *K*_i_ of the lead compound siramesine. Binding affinities of FA4 were also measured by flow cytometry in the pancreatic cancer cells in which the FA4 cytotoxic effect was investigated, revealing similar low nanomolar IC_50_ values in all the cells (IC_50_ values ~ 10 nM). Because the FA4 structure mimics that of siramesine, we also measured the binding affinity of FA4 for the sigma-1 receptor, as we found that siramesine binds the two sigma subtypes equally well [8]. Despite that a 5-fold lower affinity of FA4 for the sigma-1 receptor compared to the sigma-2 receptor was found (IC_50_ values ~ 50 nM), implication of the sigma-1 receptor in the overall activity, although marginal, cannot be ruled out.

Except for the murine PANC02 cells, FA4 exhibited a 3- to 8-fold more potent cytotoxic activity compared to that of known thiosemicarbazones (MLP44, PS3 and ACthio-1) in all the cancer cell lines investigated. Worthy of note is the activity of FA4 in PANC-1, an aggressive cell line of clinical importance that eventually develops resistance to gemcitabine [13]. Indeed, while other sigma-2 ‘pure’ ligands (devoid of metal chelation activity) [3] and the ‘pure’ metal chelator thiosemicarbazone ACthio-1 (devoid of sigma-2 affinity) did not exhibit any cytotoxicity in PANC-1 cells, the synergistic effect of the multifunctional agents seems to be a strategy in this cell line. Indeed, thiosemicarbazones carrying the sigma-receptor targeting basic moiety (PS3, MLP44, FA4) exert cytotoxicity in PANC-1 cells. In our hands, the best results were so far reached by the sigma-2 targeting thiosemicarbazones MLP44 and PS3, but FA4 performed better, with a more potent activity in PANC-1 cells and a lower cytotoxicity in non-tumor cells. Although HPDE cells are immortalized and do not exactly recapitulate non-transformed epithelial pancreatic cells, these data provide an indication of a selective activity of FA4 towards cancer cells rather than non-transformed cells. An important cytotoxic activity compared to the other thiosemicarbazones was also shown by FA4 in the MiaPaCa2 cell line, another widely used pancreatic cancer model, although less aggressive and more sensitive to gemcitabine than PANC-1 [13].

The encouraging results from the cytotoxicity assays, that demonstrate a ‘superior’ activity of FA4 in pancreatic cancer cells compared to the previously generated thiosemicarbazones, prompted us to evaluate the possible apoptotic pathways induced by the four thiosemicarbazones (FA4, MLP44, PS3 and ACthio-1) in our panel of diverse pancreatic cancer cells. The analysis of ER-dependent (ER stress sensors, caspase 7/caspase 3 axis) and mitochondria-dependent (mitochondrial ROS and depolarization, caspase 9/caspase 3 axis) pathways revealed variable activation of the apoptotic pathways dependent on ER and mitochondria, that in turn depended on the cell line used. The variegate results show that different pancreatic cancer cells treated with the same compound may activate cell death pathways to different extents and with different prevailing mechanisms. It is also clear that modification of the basic moiety in these thiosemicarbazones leads to activation of diverse pathways, dependent on ER or mitochondria. Noteworthy, FA4 induced apoptosis and ER stress in all cell lines tested, except MiaPaCa2 cells, when used at its IC50 concentration, which is in the low micromolar range. Although obtained *in vitro*, this result indicates a promising cytotoxic potential of FA4 at concentrations that could be reached in preclinical models or even in clinical settings. The differential sensitivity of pancreatic cancer cell lines to FA4 and other thiosemicarbazones may be due to several interconnected factors, including the reactivity of ER stress mechanisms, the vulnerability of mitochondria to ROS-induced damage and the activity of autophagy.

Notably, while the other thiosemicarbazones had a variable effect on the activation of ER- or mitochondria-dependent pro-apoptotic axis, FA4 activated both pro-apoptotic pathways in each cell line except for MiaPaCa2. The opposite behavior of MiaPaCa2 and the other cell lines indicates that the differences in the genotype and biochemical pathways of each cell line may affect the ability of slightly different thiosemicarbazones to drive or prevent pro-apoptotic pathways. At least two differences emerged between MiaPaCa2 and the other cell lines. First, in MiaPaCa2 cells FA4 did not induce any increase in mitochondrial ROS that were the *primum movens* of apoptosis induced by this thiosemicarbazone, according to the protective role of mitoQ. Second, we noticed different expression levels of authophagosome proteins and sequestration markers. This observation may have a relevant biological meaning because autophagy has been linked to the apoptotic mechanism activated by sigma-2 receptors. Importantly, pancreatic cancer cells less prone to activate autophagic pathways are less responsive to therapy [32]. Our finding that autophagic proteins are less abundantly expressed in MiaPaCa2 cells compared to the other cell lines tested suggests that MiaPaCa2 cells may be less reactive to cell death mechanisms induced by ER stress and mitochondrial damage elicited by FA4. On the other hand, it is known that autophagy may play either a pro-tumor or an anti-tumor effect in pancreatic cancer cells [33]. Intriguingly, we found that FA4 reduced the expression of specific autophagosome proteins and sequestration markers in all responsive cell lines, but not in MiaPaCa2. We hypothesize that FA4 prevents the protective/anti-tumor effect of autophagy in pancreatic cancer cells. By contrast, FA4-unresponsive MiaPaCa2 cells, which exhibit a low and non-tunable autophagy, are protected from FA4. These results suggest that increases in mitochondrial ROS and/or decreases in specific autophagic markers induced by FA4 are important in amplifying the cytotoxicity of FA4 following ER stress and mitochondrial damage.

The promising cytotoxicity recorded *in vitro* prompted us to evaluate how FA4 performs in xenografts *in vivo*. We found that also at a lower concentration FA4 was able to significantly reduce tumor volumes compared to control and gemcitabine treatment. The effects observed in xenografts recapitulated the viability data observed *in vitro*. Indeed, FA4 effectively reduced the growth of PANC-1 tumors, but not of MiaPaCa2 tumors. This result is of particular interest because PANC-1 xenografts were more resistant to gemcitabine than MiaPaCa2 xenografts. We recognize that we only compared one FA4-sensitive/gemcitabine resistant pancreatic tumor and one FA4-resistant/gemcitabine sensitive pancreatic tumor, but according to our data, we speculate that FA4 may be proposed as an alternative to gemcitabine in tumors unresponsive to the first line treatment. At short term, i.e., after 15 days of treatment, we could not detect tumor regression in PANC-1 xenografts treated with FA4, but only a significant delay in tumor growth, followed by a cytostatic effect when animals were treated with a higher dosage of FA4. Since apoptosis dependent on ER stress and mitochondria depolarization is not the only mechanisms that can induce tumor regression, we hypothesize that other driving factors, not affected by FA4, continue to stimulate tumor growth. Alternatively, we cannot exclude that our treatment was too short to elicit a pronounced apoptotic effect of FA4, able to determine tumor regression. To clarify this point and to deepen the safety profile of FA4, we doubled the time of treatment of PANC1 xenografts up to 30 days. Under these conditions, the efficacy of FA4 was increased, i.e., the low dosage produced a cytostatic effect, while the high dose induced a tumor regression effect, likely due to strong intratumor apoptosis. These data suggest that FA4 is a well-tunable agent, able to exert either cytostatic or cytoreductive effects depending of the time and dosage chosen. Importantly, no signs of toxicity were recorded during and after treatment. In the xenograft experiments, we used two concentrations (750 nM and 1.5 µM) that were below the IC50 of FA4 (3.01 µM) in PANC1 cells. When used at the same concentration of its IC50, FA4 activated the key mechanisms related to its cytotoxic effect, i.e., ER stress-dependent and mitochondrial damage-dependent apoptosis in PANC1 cells. FA4-treated tumors recapitulated these events, as suggested by increased intratumor active caspase 3 and by increased lipid peroxidation, indicative of oxidative damage. These data suggest that the cytotoxic mechanisms observed *in vitro* also occur intratumorally *in vivo*.

In conclusion, through the use of metal chelator thiosemicarbazones targeting sigma receptors, (FA4, MLP44, and PS3) and ACthio-1 (metal chelator), we have shown how small differences (i.e. diverse basic moiety) in the structure of thiosemicarbazone congeners can lead to the activation of different pathways in the same cell types. Importantly, we found that in different cells different pathways are affected when treated with the same compound. These differences should be taken into account in the perspective of personalized medicine-based approaches with therapies that can more efficiently target the specific characteristics of each tumor type. Additionally, the presence of the sigma-2 receptor targeting moiety may result in a more specific tumor delivery, given the higher density of these receptors in pancreatic tumors. Last but not the least, we noted that FA4 provided promising antitumor activity in the aggressive PANC-1 preclinical tumor model, that is resistant to gemcitabine and is a prototypical example of pancreatic cancer that urgently needs novel treatment options. Our work highlights the potential of FA4 as a possible monotherapy against pancreatic cancers unresponsive to gemcitabine, as an alternative to the pharmaceutical strategies currently in use. Together, the results obtained warrant further studies to define the FA4 profile in patient-derived pancreatic cancers, particularly for those that, like PANC-1, are unresponsive to conventional chemotherapy.

## Supplementary Information

ESM 1 Chemistry: experimental and Scheme S1; Hematochemical parameters of treated animals in Table S1; Density of sigma receptors in pancreatic cells by flow cytometry studies in Figure S1; Activation of caspase 3, 7, 9 in tumor pancreatic cells by FA4 administered at its IC50 values in Figure S2; Immunoblot of GRP78, ATF6, IRE1 and PERK in tumor pancreatic cells treated with FA4 at its IC50 values in Figure S3; NADPH oxidase, superoxide dismutase 1 and catalase, activities in tumor pancreatic cells treated with FA4 in Figure S4; Growth of PANC-1 and MiaPaCa2 xenografts treated with FA4, in Figure S5. (DOCX 1.08 MB)

## Declarations

The animal care and experimental procedures were approved by the Bio-Ethical Committee of the Italian Ministry of Health (#122/2015-PR).
