# Supplementary material for: Multifunctional thiosemicarbazones targeting sigma receptors: in vitro and in vivo antitumor activities in pancreatic cancer models
Source: Cell Oncol (Dordr). 2021 Sep 29;44(6):1307–23. doi: 10.1007/s13402-021-00638-5 (PMC8648660; doi:10.1007/s13402-021-00638-5)

**Supplementary Information**

**Multifunctional thiosemicarbazones targeting sigma receptors: *in vitro* and *in vivo* antitumor activity in adenocarcinoma pancreatic models**

Mauro Niso^a#^, Joanna Kopecka^b#^, Francesca Serena Abatematteo^a^, , Francesco Berardi ^a^, Chiara Riganti^b*^, Carmen Abate^a*^

^a^Università degli Studi di Bari ALDO MORO, Dipartimento di Farmacia-Scienze del Farmaco, Via Orabona 4, 70125 Bari.

^b^Department of Oncology, University of Turin, via Santena 5/bis, 10126, Torino, Italy

^#^Equally Contributing Authors

^*^Corresponding Authors

Chiara Riganti, via Santena 5/bis, 10126, Torino, Italy, +390116705857, chiara.riganti@unito.it

Carmen Abate, via Orabona 4, 70125, Bari, Italy, +390805442231, [carmen.abate@uniba.it](mailto:carmen.abate@uniba.it)

**Table of Contents (total of 6 pages)**

Chemistry, page S2, S3;

Scheme S1, page S4;

Table S1, Hematochemical parameters of treated animals, page S5;

Figure S1, Density of sigma receptors in pancreatic cells by flow cytometry studies, page S6.

Figure S2, Activation of caspase 3, 7, 9 in tumor pancreatic cell lines by **FA4** administered at its IC50 concentration, page S7;

Figure S3, Immunoblot of GRP78, ATF6, IRE1 and PERK in tumor pancreatic cell lines treated with **FA4** administered at its IC50 concentration, page S8;

Figure S4, Pro-oxidant (NADPH oxidase) and anti-oxidant (superoxide dismutase 1, catalase) enzymes’ ativities in tumor pancreatic cells treated with **FA4**, page S9.

Figure S5: Growth of PANC-1 and MiaPaCa2 xenografts treated with **FA4**, page S10.

**Chemistry**

Column chromatography was performed with 60 Å pore size silica gel as the stationary phase (1:30 w/w, 63−200 μm particle size, from ICN). Melting points was determined in open capillaries on a Gallenkamp electrothermal apparatus. Purity of **FA4** was established by high-performance liquid chromatography (HPLC) on an Agilent Infinity 1260 system equipped with diode array with a multiwavelenght UV/vis detector set at λ = 230 nm, 254 nm and 280 nm, through a Phenomenex Gemini RP-18 column (250 × 4.6 mm, 5 μm particle size, MeOH/H_2_O, 80:20 v/v at a flow rate of 0.8 mL/min). ^1^H NMR spectra were recorded on a 500-vnmrs500 Agilent spectrometer (499.801 MHz). The following data were reported: chemical shift (δ) in parts per million (ppm), multiplicity (s = singlet, d = doublet, t = triplet, m = multiplet), integration, and coupling constant(s) in hertz. Mass spectrum was recorded on an Agilent 6890-5973 MSD gas chromatograph/mass spectrometer. High resolution mass spectroscopy (HRMS) was performed on a Agilent 6530 Accurate-Mass Q-TOF LC/MS spectrometer. Chemicals were from Aldrich, and were used without any further purification.

**1-[4-(3*H*-spiro[isobenzofuran-1,4’-piperidine]-1’-yl)butyl]indoline-2,3-dione. (2)**

A solution of **1** (0.297 g, 1.25 mmol) in CH_3_CN (10 mL) was added with K_2_CO_3_ (0.143 g, 1.04 mmol) and 3*H*-spiro[isobenzofuran-1,4’-piperidine (0.196 g, 1.04 mmol). The resulting mixture was stirred under reflux overnight. The solvent was then removed under reduced pressure, and the residue was taken up with H_2_O and extracted with CH_2_Cl_2_ (3 × 7 mL). The collected organic layers were dried (Na_2_SO_4_) and evaporated under reduced pressure to afford a crude dark-red oil which was purified by column chromatography (AcOEt/MeOH 9:1) to give the title compound. GC/MS *m*/*z* 390 (M^+^, 10), 362 (15), 202 (100). The free base, dissolved in CH_2_Cl_2_ was transformed into the corresponding hydrochloride salt by addition of a solution of Et_2_O saturated with gaseous HCl. QTOF-HRMS for C_24_H_26_N_2_O_3_ (*m*/*z*): [M+H]^+^ calcd, 391.2022; found, 391.2021; [M+Na]^+^ calcd, 413.1841; found, 413.1832.

**(*Z*)-2-(1-(4-(3*H*-spiro[isobenzofuran-1,4’-piperidine]-1’-yl)butyl)-2-oxoindolin-3-ylidene)-*N*,*N*-dimethylhydrazinecarbothioamide hydrochloride. (FA4)** 4,4-Dimethyl-3-thiosemicarbazide (0.017 g, 0.14 mmol) was added to a solution of **2** (0.055 g, 0.13 mmol) in hot ethanol and the mixture was refluxed for 5h. Upon cooling, a solid was obtained, filtered and washed with cold EtOH. Crystallization from EtOH (absolute) provided the title compound as yellow crystals (0.048 g, 70% yield), mp = 203-204 °C; ^1^H NMR (500 MHz, CD_3_OD) δ 1.80-1.90 (m, 4H), 1.92-1.98 (m, 2H), 2.25-2.35 (m, 2H), 3.15-3.25 (m, 2H), 3.40 (m, 4H), 3.48 (s, 6H), 3.93 (t, 2H, *J* = 6.4 Hz), 5.10 (s, 2H), 7.14-7.22 (m, 3H), 7.27-7.36 (m, 3H), 7.43 (dt, 1H, *J_1_* = 7.8 Hz, *J_2_* = 1.5 Hz), 7.88 (br s, 1H, NH), 7.90-7.93 (m, 1H), 8.42 (s, 1H); QTOF-HRMS for C_24_H_26_N_2_O_3_ (*m*/*z*): [M+H]^+^ calcd, 492.2433; found, 492.2434.

**Scheme S1**

Reagents: A) 3*H*-spiro[isobenzofuran-1,4’-piperidine, K_2_CO_3_, CH_3_CN, Δ; B) 4,4-Dimethyl-3-thiosemicarbazide, EtOH, Δ.

**Table S1.** Hematochemical parameters of treated animals

|  | Ctrl | FA4 *^low^* | FA4 *^high^* | GEM |
| --- | --- | --- | --- | --- |
| RBC (x 10^6^/µl) | 14.09±1.937 | 12.67±2.39 | 13.26 ±2.37 | 11.28±0.98 |
| Hb (g/dl) | 13.98±1.18 | 12.87±3.43 | 12.83.2-83 | 11.03±1.94 |
| WBC (x 10^3^/µl) | 15.69±3.48 | 14.38±3.95 | 16.07±3.59 | 12.18±2.39 |
| PLT (x 10^3^/µl) | 984±302 | 983±283 | 1192±334 | 931±165 |
| LDH (U/l) | 6594±1294 | 6453±604 | 7539±506 | 6704±832 |
| AST (U/l) | 103±29 | 132±48 | 115±29 | 142±18 |
| ALT (U/l) | 39±10 | 35±7 | 38±11 | 36±12 |
| AP (U/l) | 107±14 | 117±32 | 162±45 | 134±43 |
| Creatinine (mg/l) | 0.078±0.014 | 0.084±0.009 | 0.075±0.010 | 0.084±0.008 |
| CPK (U/l) | 384±44 | 376±81 | 309±34 | 309±32 |

Mice were treated as described in Figure 6. Blood was collected immediately after euthanasia and analyzed for red blood cells (RBC) count, hemoglobin (Hb), white blood cells (WBC) count, platelets (PLT) count, lactate dehydrogenase (LDH), aspartate aminotransferase (AST), alanine aminotransferase (ALT), alkaline phosphatase (AP), creatinine, creatine phosphokinase (CPK). Data are presented as means ± SD.

**Figure S1.** Density of sigma receptors in pancreatic cells by flow cytometry studies. Results are means ± SEM (n = 3), P < 0.05.

**A.** Density of sigma-2 receptor in pancreatic cells

**B.** Density of sigma-1 receptor in pancreatic cells

**Figure S2.** Activation of caspase 3, 7, 9 by **FA4** in pancreatic cell lines. Fluorimetric measure of caspase 3, 7, 9 in cells treated 2 h with a concentration of **FA4** corresponding to its IC50 in each cell line (see Table 2). Results are means ± SEM (n = 3), P < 0.05.

| **A** | **B** | **C** |
| --- | --- | --- |
| *MiaPaca2* |  |  |
|  |  |  |
| *PANC-1* |  |  |
|  |  |  |
| *PANC02* |  |  |
|  |  |  |
| *KP02* |  |  |
|  |  |  |
| *AspC1* |  |  |
|  |  |  |

**Figure S3.** Immunoblot of GRP78, ATF6, IRE1 and PERK in the indicated cell lines treated 2 h with a concentration of **FA4** corresponding to its IC50 in each cell line (see Table 2). The image is representative of three independent experiments. Tubulin was used as control of equal protein loading.

**
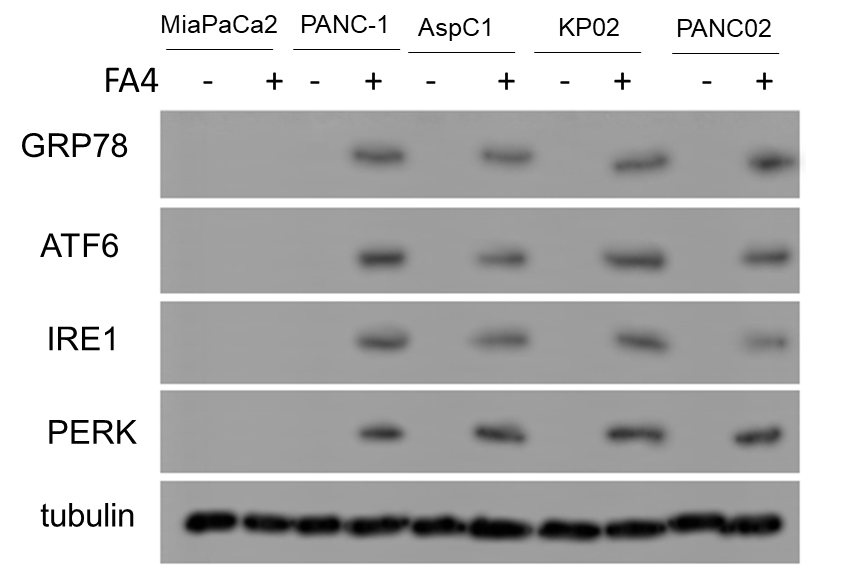
**

**Figure S4.** Activities of pro-oxidant NADPH oxidase (A) and anti-oxidant superoxide dismutase 1 (B), and catalase (C) enzymes in cells treated treated 2 h with 50 µM **FA4**. Results are means ± SEM (n = 3).

| **A** | **B** | **C** |
| --- | --- | --- |
| *MiaPaca2* |  |  |
|  |  |  |
| *PANC-1* |  |  |
|  |  |  |
| *PANC02* |  |  |
|  |  |  |
| *KP02* |  |  |
|  |  |  |
| *AspC1* |  |  |
|  |  |  |

**Figure S5. FA4 efficacy against PANC-1 and MiaPaCa2 xenografts.** FA4 in C57BL/6 mice bearing PANC-1 (panel A) or MiaPaCa2 (panel B) tumors, treated for 15 days as follows: 1) Vehicle group (black line, 100 µL saline solution); 2) FA4*^low^* group (red line, 750 nmoles FA4 in 100 µL saline solution); 3) FA4*^high^* group (yellow line, 1500 nmoles FA4 in 100 µL saline solution); 4) Gemcitabine group (green line, 20 mg/kg gemcitabine, twice a week). Animals were euthanized at day 18. Results are means ± SEM (n = 8). ***P<0.001: FA4-groups *vs* vehicle (day 18); ^#^P<0.05, ^##^P<0.001; FA4-groups *vs* gemcitabine (day 18).


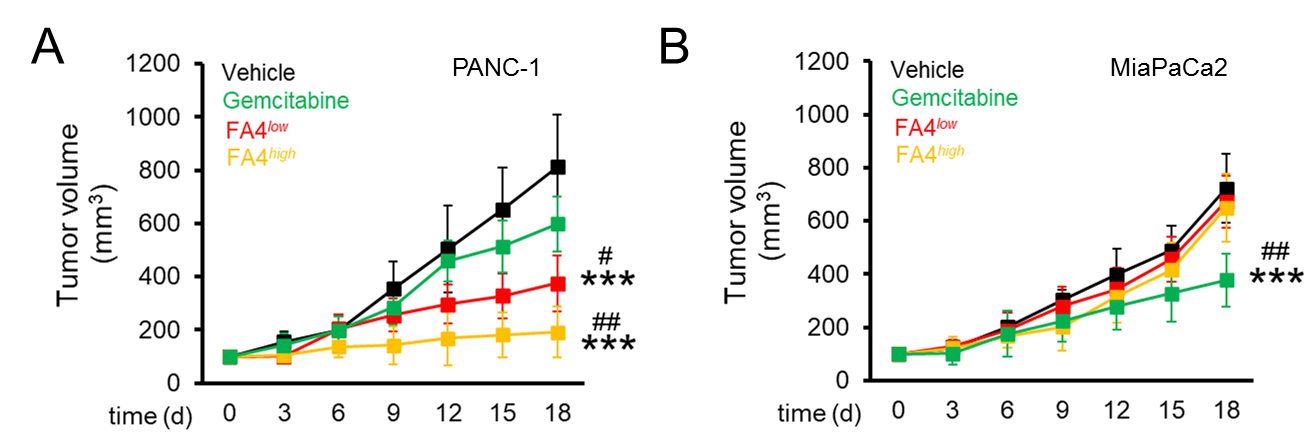

Supplement: Supplementary file 1 — Chemistry: experimental and Scheme S1; Hematochemical parameters of treated animals in Table S1; Density of sigma receptors in pancreatic cells by flow cytometry studies in Figure S1; Activation of caspase 3, 7, 9 in tumor pancreatic cells by FA4 administered at its IC50 values in Figure S2; Immunoblot of GRP78, ATF6, IRE1 and PERK in tumor pancreatic cells treated with FA4 at its IC50 values in Figure S3; NADPH oxidase, superoxide dismutase 1 and catalase, activities in tumor pancreatic cells treated with FA4 in Figure S4; Growth of PANC-1 and MiaPaCa2 xenografts treated with FA4, in Figure S5. (DOCX 1.08 MB) [file 13402_2021_638_MOESM1_ESM.docx]
